# Hyaluronic acid fat graft myringoplasty: how we do it

**DOI:** 10.1111/j.1749-4486.2008.01823.x

**Published:** 2008-12

**Authors:** I Saliba

**Affiliations:** Department of Otolaryngology – Head and Neck Surgery, Centre Hospitalier de l’Université de Montréal (Pavillon Hôtel Dieu), Montreal UniversityMontreal, QC, Canada

Dear Editor,

Tympanic membrane (TM) perforation is most commonly a result of infection, trauma, or the sequelae of tympanostomy tube insertion. Although 88% of traumatic perforations of any size heal without intervention, the remainders become chronic and require treatment.^1^ Without closure, morbidity may include hearing loss, chronic otorrhea and cholesteatoma formation.^2^ These tympanic membrane perforations can be repaired surgically; however, doing so requires a general anesthesia which is often protracted and/or hypotensive, and which can represent an unacceptable risk in older patients.^3^ Day-stay surgery has become an integral part of modern otolaryngology. Myringoplasty under local anesthesia is a short, simple, cost-effective and minimally invasive technique compared with traditional myringoplasty.^4^

The purpose of this prospective clinical trial was to evaluate the success rate of the Hyaluronic Acid Fat Graft Myringoplasty (HAFGM) performed under local anesthesia as an outpatient clinic procedure and to discuss the utilities and advantages of this new procedure described by the author.

## Materials and methods

fThis study was conducted at the outpatient clinic of the Otolaryngology department at the Montreal University Hospital Center (CHUM) – Hotel Dieu de Montréal, a tertiary care center between January 2007 and January 2008. Twenty one consecutive patients with persistent tympanic membrane perforation were included in this study. All patients underwent HAFGM and responded to these criteria: (i) perforations were central, (ii) present for at least 6 months, (iii) without evidence of active chronic otitis media, cholesteatoma or retraction pocket formation, (iv) without suspected ossicular pathology on microscopic examination and (v) air-bone gap was of 45 dB or better. The size of the perforation was not considered an exclusion criterion. Perforations were divided into four groups upon their sizes: small perforation (group I), medium perforation (group II), large perforation (group III) and total perforation (group IV) (Table 1). There were four patients in the group I, nine patients in the group II, six patients in the group III and two patients in the group IV. Hearing improvement was assessed using the audiogram results obtained preoperatively and at least 4 months postoperatively. Hearing parameters used were the change in air–bone gap. Air–bone gap was calculated as the average difference between air conduction and bone conduction at 0.5, 1, 2 and 4 kHz. The first post-operative appointment was scheduled at 2 month or before if there is a complication. The follow up was done at 4–6 and 12 months post procedure and then on a 6 month basis for 2 years period. All patients were fully informed about the description of the HAFGM procedure and gave their informed consent after a discussion of the alternatives that included doing nothing or performing a traditional myringoplasty. This was approved by our institutional review board.

**Table 1:**
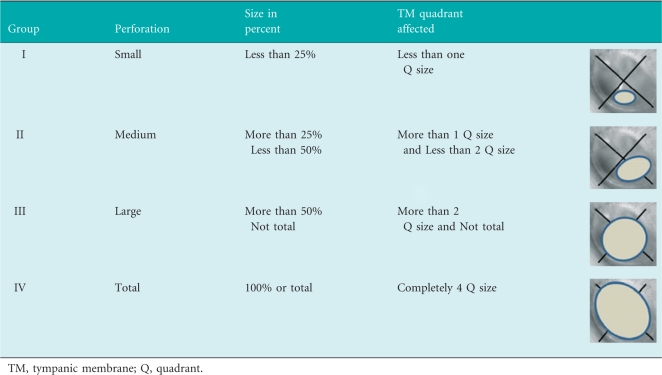
Perforation size subdivision based on the four tympanic membrane quadrant size (Saliba’s subdivision)

### Product description

**Fig. 1:**
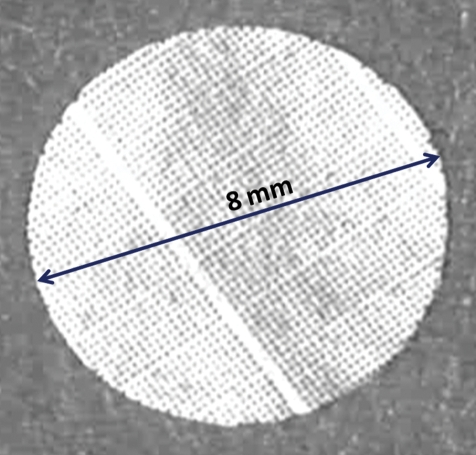
Epidisc otologic lamina composed of hyaluronic acid; 8 mm diameter.

Epidisc otologic lamina is a biomaterial composed of hyaluronic acid ester (Epidisc otologic lamina; Xomed-Medtronic, Jacksonville, FL, USA) a naturally occurring constituent of the extracellular matrix. The 8 mm diameter transparent lamina has microperforations to allow permeability facilitating drainage of exudates at the surgical site (Fig. 1).

### Surgical technique

**Fig. 2:**
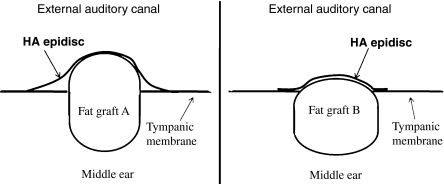
Fat graft position and hyaluronic acid (HA) epidisc position and its relation to the tympanic membrane remnant and to the fat graft. In fat graft A: Incorrect position of the fat graft as well as of the HA epidisc. In fat graft B: Correct position of the fat graft as well as of the HA epidisc.

At the outpatient clinic, the patients’ ears were prepared. The external auditory canal and the region below the mastoid tip and behind the sternocleidomastoid muscle were infiltrated with 1 percent lidocaine with 1:100 000 epinephrine. A 5 mm incision is made at this level. Care was taken to harvest a sufficient sample of fat which was at least twice the size of the perforation. The incision is closed with two absorbable sutures. After the perforation’s margins were de-epithelialized circumferentially, gelfoam pieces were placed into the middle ear through the perforation to support the fat graft in the cases of medium, large and total perforation but not in small sized one. The fat graft was then inserted through the perforation as an hourglass shaped plug. The lateral fat bulging should not be too high. Care should be taken to get an intimate contact between the epidisc, the fat graft and the tympanic membrane. The epidisc should carefully overlap, even if in a minimum extent, all the intact epithelium edge around the perforation (Fig. 2). If the malleus was denuded by the perforation, the malleus handle was surrounded by the fat graft. Depending on the TM perforation size, one or two HA epidisc are placed over the fat graft. In the case of total perforation, the HA epidisc cover the fat graft and the medial edge of the external auditory canal skin near the annulus. The HA is then covered with a pieces of gelfoam soaked with saline and the ear canal is filled with bacitracin/polymyxin ointment. It is recommended to avoid excessive pressure on the HA epidisc. No other ear dressing was required. These gelfoam pieces were used to prevent the displacement of the HA and to support the fat graft from both sides. Patients were discharged immediately after the procedure and instructed to keep their ears dry, to avoid plane travelling and to prevent strong nose-blowing for 2 months. No medication was recommended. The remaining of gelfoam pieces in the ear canal were removed at the end of the second month, the date of the first post procedure appointment. The reported surgical procedures were performed by a single surgeon (the author).

## Results

**Fig. 3:**
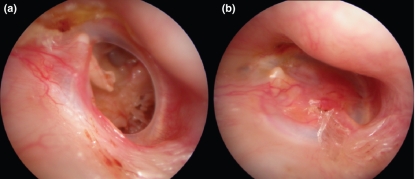
Right ear. (a) Central large perforation. (b) 2 months post hyaluronic acid fat graft myringoplasty. We notice the neovascularization of the new epithelium developed on the fat graft.

There were 13 females and eight males with the age range of 16–80 years (mean age 53.6 years). No patient had a bilateral perforation. There were nine right ears and 12 left ears. Global successful closure of the perforation was observed in 17 of 21 patients (81%). Closure rates of the perforation in group I, II, III and IV were 75%, 78%, 83% and 100% respectively. The procedures were well tolerated by all patients without major side effects or complications. There were four unsuccessful repairs of tympanic membranes of which two belong to a postoperative graft infection and two belong to an anatomic factor. In these later cases, the anterior wall of the external auditory canal presents a posterior bulging and prevents a complete identification of the perforation’s anterior rim. One patient in the group IV necessitates two procedures at 2 months interval. After the first intervention, this total perforation was closed by 80%. Total healing occurred after the second procedure. The follow-up ranged from 4 to 16 months with a mean time follow-up of 11 months. The fat graft loses 50% of its bulging at the postoperative second month (Fig. 3) and the remaining loses 45% at the postoperative fourth month. At 6-month postoperatively, we find a small stain of the fat graft in the tympanic membrane thickness. The mean preoperative air-bone gap was 22 dB. The mean air–bone gap improvement for the operated ears was 17 dB (Table 2). The mean time of the procedure is 10 min.

**Table 2:** Auditory air–bone gap outcome and changes of bone and air conduction as a percentage of patients

			Percentage						
Air–bone gap (dB) 0.5, 1, 2, and 4 kHz	Mean	SD	≤0	1–10	11–20	21–30	31–40	41–50	50+
Pre-operative	22	12	0	29	28	14	24	5	0
Post-operative	5	3	5	76	19	0	0	0	0
			Percentage change						
			dB better			dB worse			
Change (Post-operative – Pre-operative) (negative values indicate better hearing)	Mean	SD	<−20	−19–−10	−9–0	1–10	11–20	21–30	30+
Bone conduction dB (0.5, 1, 2, 4 kHz)	−0.14	0.6	0	0	100	0	0	0	0
Air conduction dB (0.5, 1, 2, 4 kHz)	−19	10	39	33	28	0	0	0	0

SD, standard deviation.

## Discussion

Ringenberg^5^ first described fat plug myringoplasty in 1962, with a success rate of 87% for small perforations. Since then, studies have shown a success rates ranging from 76% to 92% in cases of small perforation.^6^,^7^ Deddens *et al.* consider TM perforation size a crucial factor. Perforations, in his series, are 5–30% of the drum surface, which would be a good prognostic factor for a fat graft myringoplasty whereas larger perforation are less successful with fat graft alone.^8^ Prior *et al.* conclude that repair of tympanic membrane perforations with hyaluronic acid ester films alone is not to be recommended. In his series, five patients were operated under general anesthesia, the edges of the perforation were freshened and a sheet of HA, trimmed to a size roughly 2 mm larger in diameter than the perforation, was tucked through the perforation. The HA had dissolved but the perforations remained the same size in all five patients. The study was subsequently aborted. To our knowledge, we are the first to report this new technique of Hyaluronic Acid epidisc associated to Fat Graft Myringoplasty.

### Healing process

In the spontaneous healing phenomenon of tympanic membrane perforation, there is a continuous centrifugal migration of the outer squamous epithelial layer; it is missing supportive matrix under the regenerating epithelial layer of a perforation, preventing the influx of reparative cells and nutrients into the area of healing.^9^ In this study, we use the fat graft as a support for epithelial cell migration stimulated by the HA. We think that HA epidisc extend a centripetal migration of epithelial layer to cover the temporary fat plugging support. That’s why an intimate contact between the HA epidisc and the intact epithelial edge around the perforation is mandatory. An excessive pressure on the HA epidisc by the lateral gelfoam dressing should be avoided; it could block the epithelial migration.

### Anatomic factor

In the two unsuccessful cases of group II, the anterior wall of the external auditory canal presents a posterior bulging and prevents a complete identification of the perforation’s anterior rim resulting to an incomplete contact between the tympanic membrane remnant and the fat graft, and to a partial adherence between the tympanic membrane remnant and the HA epidisc. On the other hand, the de-epithelialization of the anterior margins’ perforation that stimulates the epithelial layer is impossible. For the future cases, we add this factor to our exclusion criteria list. These patients need an external auditory canalplasty to identify the anterior margin of the perforation and a traditional myringoplasty. Adding this anatomic factor to the exclusion criteria can improve the global successful rate of HAFGM to reach 91%. The closure rate of the perforation in group II becomes 100%. The success rate of overlay technique tympanoplasty varies from 91% to 97%. Underlay grafts success in 88% to 91%. Whereas the success rate with over-under tympanoplasty technique varies from 90% to 94%.^10^ If we respect the exclusion criteria, HAFGM may be an alternative treatment to traditional myringoplasty even for large or total perforation.

### To improve results

In a way to decrease the rate of graft infection, we suggest to soak gelfoam pieces with ofloxacine drops instead of saline and to give for all patients an oral, first generation cephalosporin antibiotic for the first postoperative week. Further study with a larger number of patients would be needed to confirm the efficacy of HAFGM for total perforation and to prove the decrease of infected graft by these two suggestions. Even though a total tympanic perforation may need two HAFGM procedures at 2 months interval, the HAFGM offers many advantages and could be considered as the first choice of treatment.

### Safety and simplicity

HA is a nonototoxic material.^11^ Sensorineural hearing loss reported in patients who had undergone tympanoplasty procedures has not been noticed in any study on fat myringoplasty^6^ neither in any of our series. Gelfoam inserted in the middle ear seems not to affect the result of conductive hearing loss at the postoperative 4th month. These pieces of gelfoam are important to prevent medialization of the fat graft.

Air–bone gap improvement for the operated ears was 17 dB and it is clinically significant. In addition to the high success rate, the HAFGM is processed under local anesthesia, in a mean time of 10 min at the outpatient clinic department.

**Keypoints**

- We evaluate the success rate of the Hyaluronic Acid Fat Graft Myringoplasty (HAFGM) performed under local anesthesia as an outpatient clinic procedure.
- Twenty one consecutive patients with persistent tympanic membrane perforation were included in this study.
- Perforations were divided into four groups upon their sizes: small (I), medium (II), large (III) and total (IV).
- The global successful rate of HAFGM reach 91%. The mean air-bone gap improvement for the operated ears was 17 dB. The mean time of the procedure is 10 min.
- The HAFGM is ideal for perforations of all sizes in all quadrants if a complete visualization of the margin is possible. There is no need for general anesthesia and also HAFGM yields high success rates better than FGM alone or than HA alone.
